# Efficacy of Microneedling Combined With Local Application of Human Umbilical Cord-Derived Mesenchymal Stem Cells Conditioned Media in Skin Brightness and Rejuvenation: A Randomized Controlled Split-Face Study

**DOI:** 10.3389/fmed.2022.837332

**Published:** 2022-05-24

**Authors:** Xuelei Liang, Jiaying Li, Yan Yan, Yongsheng Xu, Xiujuan Wang, Haixuan Wu, Yi Liu, Linfeng Li, Fenglin Zhuo

**Affiliations:** ^1^Department of Dermatology, Beijing Friendship Hospital, Capital Medical University, Beijing, China; ^2^Department of Dermatology, Xuanwu Hospital, Capital Medical University, Beijing, China

**Keywords:** microneedling, human umbilical cord-derived mesenchymal stem cells, conditioned media, skin rejuvenation, skin brightness, efficacy

## Abstract

**Background:**

Fighting skin aging signs is one of the major challenges of the 21st century, recently, mesenchymal stem cells (MSCs) and microneedling (MN) have been applied for anti-aging. This study aims to evaluate the efficacy of the combination of MN and human umbilical cord-derived mesenchymal stem cells conditioned media (hUC-MSCs-CM) in skin brightness and rejuvenation.

**Methods:**

Thirty volunteers with facial skin aging were recruited for the randomized, controlled split-face study. The left and right sides of the face were randomly applied with saline *via* MN or hUC-MSCs-CM *via* MN. Five sessions were performed for each volunteer at 2-week intervals. Two dermatologists evaluated the clinical improvement, in terms of skin brightness and texture. A satisfaction score based on a self-evaluation questionnaire was recorded at 2 weeks after the last treatment. The objective evaluation was recorded before the first treatment, and at 2 weeks after the last treatment.

**Results:**

Twenty-eight volunteers with a mean (SD) age of 41 (6.54) years old completed the trial. The investigator’s assessment for skin brightness and texture, and the self-satisfaction score revealed statistically better effects in hUC-MSCs-CM -plus-MN group than in MN alone (MN saline) group. No severe side effects were reported during the whole study period. Compared to MN alone group, the objective assessment revealed significant improvements in skin brightness (reduced melanin index, ultraviolet spots, and brown spots) and skin texture (reduced wrinkles and pores, and increased skin elasticity) in hUC-MSCs-CM-plus-MN group, while there were no obvious differences in skin hydration, *trans-*epidermal water loss, and the erythema index.

**Conclusion:**

The combination of hUC-MSCs-CM and MN exhibite anti-aging efficacy, and this could be used for facial rejuvenation in the future.

## Introduction

Skin aging is subject to both intrinsic (chronological) and extrinsic (environmental) factors, resulting in poor appearance and loss of functional capacity. Rejuvenating skin by fighting aging signs, such as wrinkles, enlarged pores, reduced resilience and irregular pigmentation, is one of the major challenges of the 21st century (1). Skin aging not only affects physiological skin function, but also has impacts on a person’s psychology and social life. As a result, several non-surgical treatments, such as oral treatments, ointments, dermabrasion, chemical peels, and laser therapy, have been developed over the past years, in order to counteract skin aging. However, these may be associated with prolonged recovery, dyspigmentation, and scarring.

Mesenchymal stem cells (MSCs) have the advantage of being easy to isolation, expansion, and multipotentiality, so they are popular in the field of skin rejuvenation therapy (2). These release several growth factors in autocrine and paracrine ways (3). There are various types of MSCs, including adipose-derived stem cells, bone marrow-derived stem cells (BMMSCs), and umbilical cord-derived mesenchymal stem cells. A recent study indicated that compared to BMMSCs-conditioned media, UC-MSCs-conditioned media (UC-MSCs-CM) can form premature adipocytes, collagen type 1 and collagen type 2 which have anti-aging effects (4).

Microneedling (MN) is a treatment widely applied for skin diseases and skin rejuvenation, due to its safety and efficacy. There are two main reasons for its medical use: (i) MN can accelerate the process of the skin’s natural healing. It penetrates the epidermis and papillary dermis (5), creating pores, and thereby triggering the skin’s repair mechanism (6). This can result in the short-term aggregation of inflammatory cells, fibroblasts proliferation, long-term remodeling, and the synthesis of collagen and elastin (5). (ii) MN can enhance the penetration of drugs (5). It is difficult for drugs to be absorbed through the skin due to the stratum corneum barrier (7). MN creates small transient holes that penetrate the stratum corneum barrier within a short period of time (8).

The present study aims to explore the synergistic effect of MN combined with hUC-MSCs-CM against aging skin. Non-invasive skin-measuring devices were used to objectively assess the changes in skin brightness and rejuvenation before and at 2 weeks after the final treatment.

## Materials and Methods

### Subjects

A total of 30 volunteers with facial photo-aging (Fitzpatrick III 20, IV 10) were included for the present study. Two participants were withdrawn from the study due to job-related factors. The remaining 28 volunteers completed the study. These volunteers were within 35–60 years old, with a mean (SD) age of 41 (6.54) years old. The exclusion criteria included obvious inflammation, ulcer or effusion on the face, and previous skin rejuvenation treatment in the past 3 months.

### Study Design

The present study was a randomized controlled split-face study. The Institutional Review Board of Beijing Friendship Hospital, Capital Medical University approved the study (Approval No. 2017-P2-086-01). All volunteers were thoroughly counseled on the potential risks and benefits before they signed the informed consent form, based on the 1975 Declaration of Helsinki. The registration number in http://www.chictr.org.cn is ChiCTR-INR-17013311.

### Randomization and Allocation Concealment

MN plus hUC-MSCs-CM (Beijing Origife Health Care Co., Ltd., China) group and MN alone group (control) were randomly allocated to the left or right face side. Each participant was assigned a number, according to the random number table created by the computer software. If the number was odd, the left side was used as the test side. If the number was even, the right side was used as the test side. To ensure allocation concealment, the doctors in MN operation wore colorful glasses which are normally used to protect the eyes from Intense pulse Light treatment in our department. While each volunteer’s eyes were covered with eight layers of sterile gauze before treatment.

### Intervention

Prior to treatment, the faces of all participants were cleaned using facial cleanser (Nivea, Nivea Co., Ltd., Shanghai, China) and anesthetized with 5% compound lidocaine cream (Beijing Unisplendour Pharmaceutical Co., Ltd., China). For one side, 1.0 ml of saline or hUC-MSCs-CM was painted and the remaining 1.0 ml was added locally after dermaroller MN treatment, which was performed in eight rows, with a total of 192 needles, 0.5 mm in length (Suzhou Cynour Photoelectric Technology Co., Ltd., China)., the same method was performed on the other side. The endpoint of treatment was the presence of uniform erythema over the face. Generally, 2 ml saline or hUC-MSCs-CM could be completely absorbed. The participants received five sessions of treatment at 2-week intervals for a total of 10 weeks. Table 1 presents the densities of the main growth factors derived from hUC-MSCs-CM, which were measured using the Human ProcartaPlex Growth Factor Panel (11 plex) (Cat. No. EPX110-12170-901, Invitrogen, Thermo Fisher, United States).

**TABLE 1:** Analysis of multiple cytokines secreted from hUC-MSCs-CM.

Included growth factors	Concentration (pg/mL), Mean (SD)
BDNF	22.65 (3.21)
NGF beta	34.94 (5.79)
EGF	6983.63 (523.88)
FGF-2	296.32 (55.78)
HGF	880.11 (95.33)
LIF	11.03 (2.76)
PDGF-BB	35.21 (1.88)
PIGF	9.25 (1.37)
SCF	25.12 (2.19)
VEGF-A	930.93 (77.21)
VEGF-D	50.68 (10.76)

*hUC-MSCs-CM: human umbilical cord-derived mesenchymal stem cells conditioned media; BDNF: Brain-derived neurotrophic factor; NGF beta: Nerve growth factor-beta; EGF: Epidermal growth factor; FGF-2: Fibroblast growth factor-2; HGF: Hepatocyte growth factor; LIF: Leukemia inhibitory factor; PDGF-BB: Platelet-derived growth factor-BB; PIGF: Placental growth factor; SCF: Stem cell factor; VEGF: Vascular endothelial growth factor.*

### Instrument Measurements

Prior to all measurements and assessments, the volunteers underwent an acclimatization period of 30 min under controlled standardized conditions (20°C, 40% humidity). Skin measurements were performed at baseline and 2 weeks after the 5th treatment. Cutometer ^®^ (MPA 580, Courage and Khazaka Electronic GmbH, Cologne, Germany) was used to test the hydration, transepidermal water loss (TEWL), elasticity, melanin index (MI), and erythema index. Photographs of both sides of the face, and the scores for skin spots, wrinkles, pores, ultraviolet spots, brown spots, and red spots were recorded by VISIA ^®^ skin analysis (Canfield Scientific, Inc., New York, NY, United States).

### Self-Evaluation Questionnaire

At 2 weeks after the last treatment, the volunteers were instructed to evaluate their satisfaction with the efficacy, taking into account the adverse effects observed on both sides of the face, using the following scale: 0 = very dissatisfied, 1 = relatively dissatisfied, 2 = slightly dissatisfied, 3 = satisfied, 4 = relatively satisfied, and 5 = very satisfied.

### Physician’s Assessments

Two dermatologists, who were blinded to the study design and treatment, evaluated the clinical improvement, and compared it to the baseline. The assessment was based on the photographs taken by VISIA ^®^ skin analysis on both sides of the face at baseline and 2 weeks after the last treatment. The changes in skin brightness and rejuvenation were assessed, and the improvement was graded, as follows: 1 = 0–25% (no or minimal improvement), 2 = 26–50% (moderate improvement), 3 = 51–75% (marked improvement), and 4 = 76–100% (excellent improvement).

### Safety Assessment

Adverse reactions were recorded by questioning and examining the subjects during the follow-up visits.

### Statistics

Statistical analysis was carried out by SPSS software 19.0 (IBM Corp., Armonk, NY, United States). Paired *t* test was used to compare the data of objective assessment between the test side and control side at every visit. The data of subjective evaluation was analyzed with the Wilcoxon signed-rank test. The measurements of both sides at baseline and the last visit were compared by paired *t* tests to see the effect of treatment. The statistically significant difference was set as *P* < 0.05.

## Results

### Subjective Evaluation From Doctors and Participants

#### Self-Evaluation Questionnaire

A total of 28 volunteers completed the study. MN plus hUC-MSCs-CM side was more satisfied than MN alone side (*P* < 0.05, Table 2).

**TABLE 2:** Participant’s overall satisfaction with the efficacy at 2 weeks after the last treatment.

(*n*)	Not satisfied	Slightly satisfied	Moderately satisfied	Satisfied	Very satisfied	*P*-value
MN alone	5	9	10	4	0	0.000*
MN plus hUC-MSCs-CM	0	5	9	9	5	

*MN: microneedling; hUC-MSCs-CM: human umbilical cord-derived mesenchymal stem cells conditioned media. Participant’s overall satisfaction scores: ranging from 0, not satisfied, to 5, very satisfied.*

*P < 0.05.

#### Clinical Assessment

**FIGURE 1:**
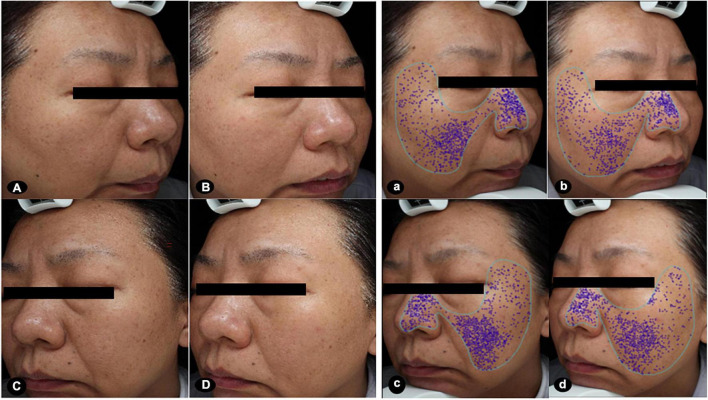
Images at baseline and after the treatments. The clinical images presented greater improvements in brightness and size of pores after MN plus hUC-MSCs-CM [**(A,a)** baseline; **(B,b)** after treatment], compared to MN alone [**(C,c)** baseline; **(D,d)** after treatment]. The score of pores at the side of MN *via* hUC-MSCs-CM decreased from 20.29 to 13.32 after treatment. While the score of pores at another side of MN alone decreased from 22.64 to 17.71 after treatment. MN: microneedling; hUC-MSCs-CM: human umbilical cord-derived mesenchymal stem cells conditioned media.

**FIGURE 2:**
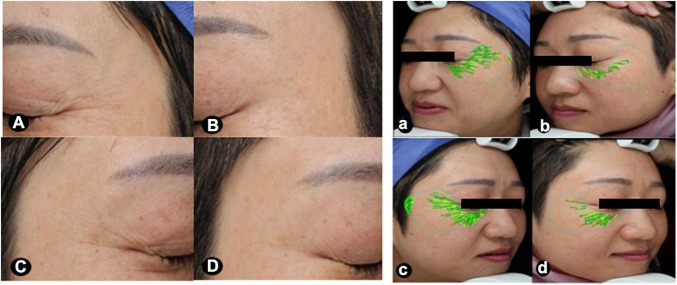
Images focusing on the periorbital areas at baseline and after treatment. The periorbital wrinkles exhibited greater improvements after MN plus hUC-MSCs-CM [**(A,a)** baseline; **(B,b)** after treatment], compared to MN alone [**(C,c)**, baseline; **(D,d)** after treatment]. VISIA ^®^ skin analysis assessment for periorbital wrinkles: a = 29.53, b = 8.39, c = 32.37, d = 15.63. MN: microneedling; hUC-MSCs-CM: human umbilical cord-derived mesenchymal stem cells conditioned media.

The physician’s global assessment of brightness and skin texture were greater improvements following hUC-MSCs-CM plus MN, compared to MN alone (*P* < 0.05, Table 3). The representative images revealed the larger improvement of pores and periocular wrinkles after MN plus hUC-MSCs-CM, compared to MN alone (Figures 1, 2).

**TABLE 3:** Physician’s global assessment at 2 weeks after the last treatment.

	No or minimal improvement	Moderate improvement	Marked improvement	Excellent improvement	*P*-value
*Physician’s global assessments for brightness (n)*					0.001*
MN alone	5	18	5	0	
MN plus hUC-MSCs-CM	0	17	11	0	
*Physician’s global assessments for skin texture (n)*					0.000*
MN alone	6	15	7	0	
MN plus hUC-MSCs-CM	2	13	7	6	

*Physician’s global assessments: grade 1: 0–25% = minimal or no improvement; grade 2: 26–50% = moderate improvement; grade 3: 51–75% = marked improvement; and grade 4: 75–100% = excellent improvement. MN: microneedling; hUC-MSCs-CM: human umbilical cord-derived mesenchymal stem cells conditioned media.*

*P < 0.05.

### Objective Assessment

At baseline, there was no statistically significant difference for skin physiological parameters, skin brightness, and skin rejuvenation in the objective assessments between MN plus hUC-MSCs-CM group and MN alone group (Table 4).

**TABLE 4:** Objective assessment at baseline.

	MN alone	MN plus hUC-MSCs-CM	*P*-value
**Skin physiological parameters**			
Hydration	62.72 (7.96)	60.66 (7.96)	0.074
TEWL	20.88 (6.55)	20.31 (6.28)	0.130
**Skin brightness**			
Melanin Index	190.65 (40.12)	196.12 (40.91)	0.076
Ultraviolet Spots	23.08 (7.58)	24.39 (7.11)	0.067
Brown Spots	39.71 (5.82)	40.21 (5.14)	0.256
Erythema Index	336.68 (60.80)	336.69 (63.08)	0.999
Red Spots	29.17 (5.93)	29.30 (5.58)	0.754
**Skin rejuvenation**			
Wrinkles	18.18 (13.38)	17.21 (13.24)	0.227
Pores	24.40 (10.77)	23.55 (10.52)	0.295
Elasticity	0.57 (0.06)	0.56 (0.07)	0.067

*MN: Microneedling; hUC-MSCs-CM: human umbilical cord-derived mesenchymal stem cells conditioned media; TEWL: Trans-epidermal water loss.*

#### Skin Physiological Parameters

Hydration and TEWL: The hydration content and TEWL are indicators for skin barrier function. There was no significant change in hydration content after treatment between MN plus hUC-MSCs-CM side [2.18 (5.80), *P* = 0.06] and MN alone side [2.07 (6.78), *P* = 0.12]. Furthermore, no significant change was found in the TEWL measurements between MN plus hUC-MSCs-CM side [1.96 (5.15), *P* = 0.05] and MN alone side [1.10 (3.79), *P* = 0.13], indicating that MN treatment did not damage the skin barrier.

#### Skin Pigmentation (Skin Brightness)

##### Melanin Index

**FIGURE 3:**
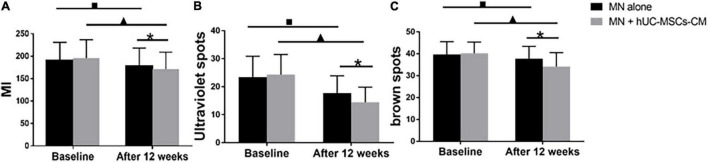
Objective non-invasive skin pigmentation measurements. **(A)** Melanin index (MI); **(B)** Ultraviolet spots; **(C)** Brown spots. MN: microneedling; hUC-MSCs-CM: human umbilical cord-derived mesenchymal stem cells conditioned media. *P < 0.05, post-treatment comparison between MN alone and MN plus hUC-MSCs-CM; ^▲^*P* < 0.05, pre-treatment and post-treatment in MN plus hUC-MSCs-CM. ^■^*P* < 0.05, pre-treatment and post-treatment in MN alone.

At 2 weeks after the last treatment, the MI decreased more in MN plus hUC-MSCs-CM group than in MN alone group [24.25 (15.55) vs. 12.36 (16.38), *P* = 0.00; Figure 3A], indicating the efficacy of hUC-MSCs-CM for skin lightening (Figures 3A, 1A,B).

##### Ultraviolet Spots

Ultraviolet spots are associated with exposure to ultraviolet sun radiation. The ultraviolet spot score of the side treated with MN plus hUC-MSCs-CM decreased from 24.39 (7.11) to 14.47 (5.38), *P* = 0.00, while the score for the side treated with MN alone decreased from 23.08 (7.58) to 17.72 (6.18), *P* = 0.00. There was no significant difference in ultraviolet spot score between the two sides before first treatment, but a significant difference was observed at the last measurement (*P* < 0.05, Figure 3B), indicating that hUC-MSCs-CM was effective in decreasing ultraviolet spots.

##### Brown Spots

The formation of brown spots is related to dyspigmentation. Compared to baseline, either one side treated with MN plus hUC-MSCs-CM and MN presented with statistically significant changes in brown spot scores 2 weeks after the final treatment. The score for MN plus hUC-MSCs-CM side decreased from 40.21 (5.14) to 34.18 (6.32) (*P* < 0.05), while the score for MN alone side decreased from 39.71 (5.82) to 37.75 (5.63) (*P* < 0.05). At the last measurement, a significant change was observed between MN plus hUC-MSCs-CM side and MN alone side (*P* = 0.00, Figure 3C), indicating that MN plus hUC-MSCs-CM has a better effect in decreasing the brown scores.

##### Erythema Index

There was no significant difference in erythema index (EI) after treatment between MN plus hUC-MSCs-CM side (*P* = 0.90) and MN alone side (*P* = 0.87). The EI score for the sides treated with MN plus hUC-MSCs-CM increased from 336.69 (63.08) at baseline to 336.85 (60.06) at 2 weeks after the final treatment. Similarly, the EI score for the sides treated with MN alone increased from 336.68 (60.80) at baseline to 337.29 (57.05) at 2 weeks following the final treatment. This indicates that MN and hUC-MSCs-CM does not induce skin inflammation.

##### Red Spots

Spots in the side treated with MN plus hUC-MSCs-CM decreased from 29.30 (5.58) to 28.20 (4.92) (*P* = 0.17), while spots on the other side decreased from 29.17 (5.93) to 28.08 (5.38) (*P* = 0.12). There was no statistically significant difference in red spot scores at the last measurement (*P* = 0.31).

### Skin Rejuvenation

#### Wrinkles

**FIGURE 4:**
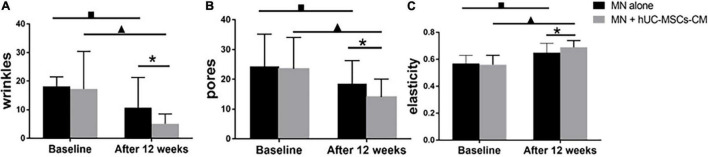
Objective non-invasive skin texture measurements: **(A)** wrinkles; **(B)** pores; **(C)** elasticity. MN: microneedling; hUC-MSCs-CM: human umbilical cord-derived mesenchymal stem cells conditioned media. *P < 0.05, post-treatment comparison between MN alone and MN plus hUC-MSCs-CM; ^▲^*P* < 0.05, pre-treatment and post-treatment in MN plus hUC-MSCs-CM; ^■^*P* < 0.05, pre-treatment and post-treatment in MN alone.

Compared to baseline, the wrinkle score for MN plus hUC-MSCs-CM side decreased to 5.06 (3.44) (*P* = 0.00), while the score for MN side decreased to 10.73 (10.63) (*P* = 0.00). At the final measurement, MN plus hUC-MSCs-CM side exhibited a more significant decrease in wrinkle measurements than in MN alone side [12.15 (10.26) vs. 7.46 (6.07), *P* = 0.00; Figures 2, 4A].

#### Pores

Significant changes were observed for pores in both MN plus hUC-MSCs-CM group (*P* = 0.00) and MN alone group (*P* = 0.00). The pores’ scores decreased from 23.55 (10.52) to 14.05 (6.11) in MN plus hUC-MSCs-CM group, while these decreased from 24.40 (10.77) to 18.59 (7.70) in MN side. Furthermore, there was a significantly better effect after treatment in hUC-MSCs-CM side, compared to MN alone side (*P* = 0.00; Figures 2, 4B), indicating that hUC-MSCs-CM can shrink the size of pores.

#### Elasticity

Compared to baseline, skin elasticity significantly improved in both groups (*P* = 0.00). At 2 weeks after the last treatment, there was a significantly higher increase in MN plus hUC-MSCs-CM group, compared to MN alone group (*P* = 0.00). Furthermore, the measurements of elasticity for MN alone group increased from 0.57 (0.06) to 0.65 (0.07), while the measurements for the other side increased from 0.56 (0.07) to 0.69 (0.05) (Figure 4C).

### Side Effects

No severe side effects were reported during the whole study period. Two participants in MN plus hUC-MSCs-CM group reported dry skin and one reported erythema. Three patients reported dry skin and two patients reported erythema in MN alone group, respectively.

## Discussion

A total of 28 volunteers with a mean (SD) age of 41 (6.54) years old completed the trial. The investigator’s assessment of skin brightness and rejuvenation, and the self-satisfaction score revealed statistically better effects in MN plus hUC-MSCs-CM group, compared to MN alone group. No severe side effects were reported during the whole study period. The objective assessments revealed significantly more improvement in skin brightness (reduced MI, ultraviolet spots, and brown spots) and skin rejuvenation (reduced wrinkles and pores, and increased skin elasticity) in MN plus hUC-MSCs-CM group, compared to MN alone group, while there were no obvious differences in skin hydration, *trans-*epidermal water loss, and erythema index.

Microneedling stimulate the dermis, which increases new collagen and elastin, thereby rejuvenating the skin, improving wrinkles and increasing skin elasticity (9). Robati RM et al. compared MN to fractional Er:YAG laser in facial skin rejuvenation, and found a comparable efficacy. However, the slight “downtime” of MN makes it preferable for many patients (10). At present, MN is widely applied for various skin diseases, particularly in the skin-of-color population (i.e., Fitzpatrick skin types IV-VI) (11). Compared to laser treatments, MN is simple and inexpensive. Furthermore, the skin barrier disruption caused by MN resolves within 72 h (12). In the present study, the skin barrier signs, including skin hydration and TEWL, had no significant difference before and after treatment, in both MN plus hUC-MSCs-CM group and MN alone group. This finding indicates the safety of MN therapy.

The combination of MN with topical agents has successfully been used in the facial rejuvenation of aged skin (13). Most recently, stem cell extracellular vesicles or cell therapies, such as adipose-derived stem cells, MSCs and BMMSCs, have been used for skin rejuvenation due to the ability to repair and regenerate tissues and organs in cosmetic and reconstructive surgeries (14, 15). The long-term safety and controllability of cell-based therapies remain controversial, making cell-derived extracellular vesicles (exosomes or conditioned medium) preferable for most therapeutic applications. The effect of conditioned media from BMMSC- or adipose-derived stem cells on skin rejuvenation has been demonstrated (16, 17). In the present study, the participant’s satisfaction score and physician’s global assessments score for facial rejuvenation were higher in MN plus hUC-MSCs-CM group, compared to MN alone group. These present results support another study, which reported that amniotic membrane stem cell-conditioned medium can rejuvenate the skin (24).

Skin brightness and texture are more correlated to skin rejuvenation. The less pigmented the skin is, the brighter it becomes. Objective pigmentation parameters, including the MI, ultraviolet spots, and brown spots, were explored in the present study. Compared to MN alone group, all three pigmentation parameters significantly decreased in combination group at 2 weeks after the final treatment. Compared to baseline, MN alone side presented with a significant decrease in MI. However, in a Korean study, the MI merely significantly decreased in subjects treated with MN combined with secretory factors of endothelial precursor cells derived from human embryonic stem cells (19). This difference may be attributed to the different length of microneedling (0.25 mm in the Korean study vs. 0.5 mm in this study). A previous study revealed that the single-session MN-treatment for melasma with a needle length of 0.5 mm resulted in significantly reduced melanin density, pendulous melanocytes, and a pathological basement membrane (20).

Except for pigmentation, the skin textures, such as wrinkles, pores and elasticity, tested by VISIA ^®^ skin analysis were also explored in the present study. Compared to baseline, wrinkles, pores, and elasticity improved in MN plus hUC-MSCs-CM group and MN alone, and the effect of the combined group was significantly better than that of MN alone group. These results can be attributed to the various growth factors of hUC-MSCs-CM, such as EGF, VEGF, PDGF-BB. For example, EGF stimulates epidermal cell proliferation (21). VEGF facilitates skin angiogenesis and increases blood vessel permeability to improve tissue nutrition. PDGF-BB is involved in fibroblast proliferation and regulates cell growth (22). According to the present findings, the changes in the skin micro-environment may lead to the greater improvement in wrinkles, pores and elasticity in combination group, compared to MN alone. A recent study concluded that the remodeling of dermal structures in skin biopsy pathology can be observed mainly on the combined side (23). Furthermore, the histometry of the epidermis revealed a significant increase in epidermal thickness on both the skin-needled and combined side (i.e., skin needling plus amniotic fluid mesenchymal stem cell derived conditioned media). This is consistent with the present results, in which the improvement in skin rejuvenation was greater in the combined side, compared to MN alone side.

In the present study, merely minor treatment-related side effects were observed. Two patients reported dry skin and one patient reported erythema in MN plus hUC-MSCs-CM group, while three patients reported dry skin and two patients reported erythema in MN alone group the dry skin was likely due to a temporary breakdown in skin barrier function caused by microneedling.

The small sample size and lack of pathological assessment were the main limitations of this study, despite the fact that great improvement was observed in the images taken by VISIA ^®^ test. However, the split—face study design presented the reliable comparison between the two groups with such a relatively small number of volunteers. In the future, a larger sample size will be considered and more objective evaluations will be applied. The repeatability and stability of hUC-MSCs-CM are always the focus when performing the project.

Up to now, only two randomized controlled trials and one control trial was published in the application of MN with and without MSCs-CM or extracts from media of MSCs in facial rejuvenation (Table 5), and these studies provide strong evidence for the effective treatment of facial aging by this method. The data from the three articles supported that combination therapy with MN *via* MSCs derivative improve significantly than MN alone.

**TABLE 5:** Facial rejuvenation studies with MN plus with MSCs.

Reference	Sample size	Methods	Results
Wang et al. (24)	30 patients	One side: the protein extracts from medium of ADSCs + MN, the other side: ultrapure water + MN. once every 2 weeks, 6 times	Compared to ultrapure water + MN, the melanin index, skin brightness, gloss, skin roughness, elasticity and wrinkles improved significantly in the protein extracts from medium of ADSCs + MN at the 12th week
Prakoeswa et al. (18)	48 females	24 women: AMSC-CM + MN the other 24 women: NS + MN once every 2 weeks, 3 times	Compared to NS + MN group, mean change of wrinkles, pores, spot-polarized and spot-ultraviolet parameters improved significantly at 8 weeks in AMSC-CM + MN group. At weeks 8, there was no improvement in skin tone
El-Domyati et al. (23)	10 patients	Right side: MN + AF-MSC-CM, left side: MN once every 2 weeks, 5 times;	collagen fibers and elastic fibers remodeling was observed mainly on the right side at 1 month after the final treatment The epidermal thickness on both sides increased similarly

*MN: microneedling; MSCs: mesenchymal stem cells; ADSCs: adipose-derived stem cells; AMSC-CM: amniotic membrane stem cell-conditioned media; NS: normal saline; AF-MSC-CM: amniotic fluid mesenchymal stem cell derived conditioned media.*

In conclusion, MN combined with hUC-MSCs-CM is a safe and effective treatment modality for facial rejuvenation, and may potentially be used as a novel method for anti-aging.

## Data Availability Statement

The raw data supporting the conclusions of this article will be made available by the authors, without undue reservation.

## Ethics Statement

The studies involving human participants were reviewed and approved by the Medical Ethics Committee of Beijing Friendship Hospital affiliated to Capital Medical University. The patients/participants provided their written informed consent to participate in this study. Written informed consent was obtained from the individual(s) for the publication of any potentially identifiable images or data included in this article.

## Author Contributions

All authors listed have made a substantial, direct, and intellectual contribution to the work, and approved it for publication.

## Conflict of Interest

The authors declare that the research was conducted in the absence of any commercial or financial relationships that could be construed as a potential conflict of interest.

## Publisher’s Note

All claims expressed in this article are solely those of the authors and do not necessarily represent those of their affiliated organizations, or those of the publisher, the editors and the reviewers. Any product that may be evaluated in this article, or claim that may be made by its manufacturer, is not guaranteed or endorsed by the publisher.
